# Lack of cross-protection against *Mycoplasma haemofelis* infection and signs of enhancement in “*Candidatus* Mycoplasma turicensis”-recovered cats

**DOI:** 10.1186/s13567-015-0240-x

**Published:** 2015-09-24

**Authors:** Julia Baumann, Marilisa Novacco, Barbara Willi, Barbara Riond, Marina L Meli, Felicitas S Boretti, Regina Hofmann-Lehmann

**Affiliations:** Clinical Laboratory, Vetsuisse Faculty, University of Zurich, Winterthurerstrasse 260, 8057 Zurich, Switzerland; Center for Clinical Studies, Vetsuisse Faculty, University of Zurich, Winterthurerstrasse 260, 8057 Zurich, Switzerland; Clinic for Small Animal Internal Medicine, Vetsuisse Faculty, University of Zurich, Winterthurerstrasse 260, 8057 Zurich, Switzerland

## Abstract

“*Mycoplasma haemofelis*” and “*Candidatus* Mycoplasma turicensis” are feline hemoplasmas that induce hemolytic anemia. Protection from homologous re-challenge was recently demonstrated in cats recovered from primary infection. Here, we determined if cats recovered from “*Cand.* M. turicensis” infection were protected against infections with the more pathogenic *M. haemofelis*. Ten specified pathogen-free cats were exposed to *M. haemofelis*. Five of the ten cats had recovered from “*Cand.* M. turicensis” bacteremia (group A), and five cats were naïve controls (group B). No cross-protection was observed. By contrast, the “*Cand.* M. turicensis”-recovered cats displayed faster *M. haemofelis* infection onset (earlier PCR-positive and anemic) than the controls. No “*Cand.* M. turicensis” was detected in any cat. *M. haemofelis* shedding was observed in saliva, feces and urine. In both groups, evidence of a Th1 response was observed (high IFN-γ, low IL-4), but IL-10 levels were also high. In group A, total, CD4+ and CD8+ T cells increased within days after *M. haemofelis* exposure. At times of maximal bacteremia, macrocytic hypochromic anemia, neutropenia, monocytosis and a decrease in leukocyte, eosinophil, and lymphocyte counts and subsets thereof (B- and T-cells, CD4+, CD8+ and CD4+CD25+ cells) were particularly significant in group A. Moreover, an increase in protein concentrations, hypoalbuminemia and a polyclonal hypergammaglobulinemia were observed. Five of ten *M. haemofelis*-infected cats subsequently cleared bacteremia without antibiotic treatment. In conclusion, the study suggests that a previous hemoplasma infection, even when the cat has ostensibly recovered, may influence subsequent infections, lead to an enhancement phenomenon and other differences in infection kinetics.

**Electronic supplementary material:**

The online version of this article (doi:10.1186/s13567-015-0240-x) contains supplementary material, which is available to authorized users.

## Introduction

Hemotropic mycoplasmas (hemoplasmas) are cell-wall free bacteria that attach to red blood cells and potentially induce hemolytic anemia [1]. In the domestic cat, at least three feline hemotropic mycoplasma species are known: *Mycoplasma haemofelis* (*M. haemofelis*), “*Candidatus* Mycoplasma haemominutum” (“*Cand.* M. haemominutum”) and “*Candidatus* Mycoplasma turicensis” (“*Cand.* M. turicensis”) [2–5]. The pathogenic potential significantly varies among the three feline species; *M. haemofelis* is the most pathogenic of the three species, and an acute infection often results in hemolytic anemia [1,6].

The diagnosis of feline hemotropic mycoplasma infection relies on the detection and differentiation of the infectious agents by sensitive and specific polymerase chain reaction (PCR) [2,7]. To quantify the humoral immune response to feline hemoplasmas, enzyme-linked immunosorbent assays (ELISA) based on a recombinant *M. haemofelis* DnaK protein have been developed [8,9]. The recombinant *M. haemofelis* protein recognizes antibodies to *M. haemofelis* and, to a lesser degree, antibodies to “*Cand.* M. turicensis” and “*Cand.* M. haemominutum”; the observed cross-reactivity of the humoral immune response led to the assumption of a potential cross-protection among the three hemoplasma infections. In a recent study, high levels of antibodies were detected in cats experimentally infected with “*Cand.* M. turicensis”, even months after the infection and bacteremia had cleared [10]. These “*Cand.* M. turicensis” PCR-negative seropositive cats were also protected from subsequent “*Cand.* M. turicensis” challenge [10,11]. Similarly, cats that had overcome *M. haemofelis* bacteremia and were PCR-negative and serologically positive for *M. haemofelis* were subsequently protected from a second *M. haemofelis* bacteremia after re-exposure [12]. Limited information is available concerning potential cross-protection against infections with different feline hemoplasma species. In some naturally infected cats and wildcats, dual or triple infections with *M. haemofelis*, “*Cand.* M. haemominutum” and “*Cand.* M. turicensis” have been reported [13–16]. However, in natural infections, it typically cannot be determined if the infections were simultaneously or sequentially acquired. Cross-protection might be expected only when the infections are sequentially acquired after an efficient immune response had been raised. Experimental co-infection was demonstrated in an early infection study using so-called large and small *Haemobartonella felis* strains [17]. However, these cats had not cleared the primary infection when superinfected with a second hemoplasma strain, and thus no cross-protection was observed [17].

A vaccination approach involving attenuated organisms may offer hemoplasma protection [12]. “*Cand.* M. turicensis” is much less pathogenic than *M. haemofelis* [2], and thus “*Cand.* M. turicensis” would be an ideal candidate for an attenuated *M. haemofelis* vaccine, if cross-protection against *M. haemofelis* exposure was found in cats that developed immunity against “*Cand.* M. turicensis”.

Thus, the aim of the present study was to investigate potential cross-protection against *M. haemofelis* in cats that had overcome a previous “*Cand.* M. turicensis” infection and to characterize the course of infection in naïve and “*Cand.* M. turicensis”-recovered cats, by analyzing hematological and clinical chemistry parameters, lymphocyte subsets, cytokine transcription levels and hemoplasma shedding patterns.

## Materials and methods

### Animals and experimental design

Ten adult male specified pathogen-free (SPF) cats (Liberty Research, Waverly, NY, USA) were included in the present study. The cats were assigned to two groups: group A comprised five cats (FIA1, FIA2, FHT1, FHX4, and FHX5) that had previously experienced experimental “*Cand.* M. turicensis” low-dose infection described in detail elsewhere [18], and group B comprised five naïve SPF cats (KCY2, ZKA2, AKL4, JCT2, and KCU1) [19]. The cats in group A were listed in the previous study as A1, A2, T1, X4 and X5 [18]. Prior to the start of this experiment, the cats in group A had overcome acute “*Cand.* M. turicensis” infection and bacteremia and were PCR-negative and serologically positive (for detailed description of the PCR and serology see below). All cats in group A were five years old, whereas in group B, three cats were three years old (KCY2, JCT2, and KCU1), and two cats were six years old (ZKA2 and AKL4). All cats had been castrated prior to this experiment.

All experiments were performed according to Swiss law and were officially approved by the veterinary office of the canton of Zurich (TVB 159/2010). The cats were kept in two separate groups in a confined university facility under barrier conditions and optimal ethological and hygienic conditions as previously described [20]. Prior to the start of the experiment, each cat was clinically examined, and the SPF status of the cats was verified by testing for absence of infections with feline hemoplasmas, parvovirus, calicivirus, coronavirus, herpesvirus-1, leukemia and immunodeficiency virus, *Bartonella henselae* and *Chlamydophila felis* using sampling procedures and methods as previously described [21].

**Figure 1:**
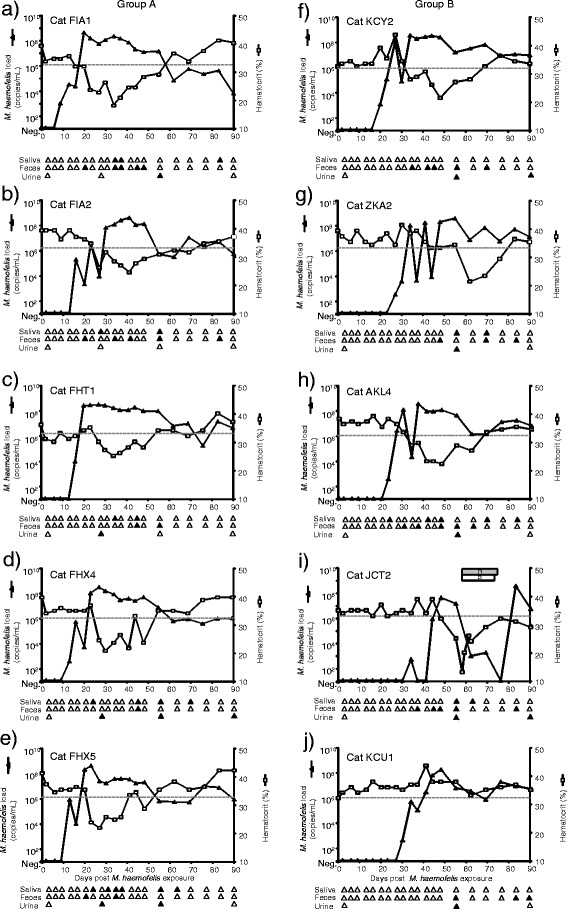
***M. haemofelis*** **loads, shedding patterns and hematocrit after** ***M. haemofelis*** **exposure in ten SPF cats.** The five cats in group A had undergone previous “*Cand.* M. turicensis” infection **(A**-**E)** and the five cats in group B were naïve control cats **(F**-**J**; the IDs for each cat are indicated in the figure**)**. The *M. haemofelis* exposure took place on day 0. The blood loads are presented as log DNA copy numbers per mL of blood (left y-axis) as determined by real-time qPCR, and the hematocrit values are given as a percentage (right y-axis). The lower reference value for the hematocrit is indicated by a dotted line. The PCR results from saliva, rectal swabs and urine are illustrated as triangles below the x-axes. The PCR-positive swabs are indicated by filled symbols; PCR-negative swabs are indicated by open symbols. For clarity, only the first 90 days post exposure are shown. The last urine sample listed under day 90 was collected on day 104. Cat JCT2 was treated with doxycycline (gray box marked “D”) and prednisolone (white box marked “P”). The data from group B have been partially previously presented [19].

All cats were subjected to low-dose *M. haemofelis* exposure on day 0 as previously described [19] (for details see below). EDTA-anticoagulated blood and serum samples were collected for PCR, hematology, clinical chemistry and serology analyses before (at day 0) and after *M. haemofelis* exposure (for time points see Figure 1 and Additional file 1). To monitor *M. haemofelis* shedding, saliva and rectal swabs were collected at the time points indicated in Figure 1 and on days 141, 232 and 286 using commercially available cotton swabs as previously described [22]. Urine samples were collected from all cats by cystocentesis at one day prior to and on days 57 and 104 after *M. haemofelis* exposure and on day 28 from the cats in group A. Urinalysis was performed, and the urine sediment was evaluated using standard procedures. Clinical condition, body temperature and body weight were recorded at each sampling time point. *M. haemofelis*-infected cats that developed severe anemia (hematocrit < 10%) and/or were in a poor general condition were treated with doxycycline orally (10 mg/kg/d, Grünenthal GmbH, Mitlödi, Switzerland) for 14 days, prednisolone orally (2 mg/kg every 12 h, gradually withdrawn, Streuli Pharma AG, Uznach, Switzerland) for ten days and fluid therapy (Ringer’s lactate solution, Fresenius Kabi (Schweiz) AG, Stans, Switzerland) (see also Figure 1I).

### Preparation of *M. haemofelis* inoculum and infection procedure

The *M. haemofelis* low-dose infection model has been previously described [19]. Briefly, heparinized infectious blood from cat QLA5 [8] preserved in dimethylsulfoxide (20%, vol/vol) in liquid nitrogen was used to prepare the inoculum. The blood sample was thawed at 37° and diluted in cold phosphate-buffered saline (PBS) to a final concentration of 1 × 10^3^ copies in 100 μL as determined by specific 16S rRNA TaqMan® real-time qPCR [14]. The inoculum was maintained on ice. Each cat was subcutaneously injected with 100 μL of the inoculum in the region of the neck within five minutes after dilution of the inoculum.

### Hematology and Coombs’ test

White blood cell differentials and complete hemograms were performed using a Sysmex XT-2000iV (Sysmex Corporation, Kobe, Japan) [23]. Hematocrit between 33% and 45% was considered within the reference range; anemia was defined as a hematocrit value below 33%. Leukopenia was defined as < 4.6 × 10^3^/μL; and neutropenia was defined as < 2.3 × 10^3^/μL. All hematological reference ranges were determined using identical methods and blood samples from 63 clinically healthy cats. Because a previous study demonstrated antibodies directed against the erythrocyte surface in some *M. haemofelis*-infected cats [24], Coombs’ test was performed. The Coombs’ test was performed towards the end of the study on day 364 post *M. haemofelis* exposure as previously described [2], with the following modifications: the feline anti-globulin Coombs’ reagent (ImmunO™, MP Biomedicals, Solon, Ohio, USA) was serially diluted from 1:2 to 1:10 240.

### Clinical chemistry

Serum chemistry was performed using a Cobas Integra 800 system (Roche Diagnostics, Rotkreuz, Switzerland) and included the laboratory’s standard feline clinical chemistry panel: bilirubin, glucose, blood urea nitrogen (BUN), creatinine, protein, albumin, cholesterol, triglyceride, alkaline phosphatase, amylase, aspartate aminotransferase, alanine aminotransferase, lipase, sodium, chloride, potassium, calcium and phosphate. Globulin values were calculated by subtraction of albumin from the total protein concentration. Reference ranges are stated as 5% and 95% quantiles and were determined for routine purposes using identical methods and blood samples from 59 clinically healthy cats.

### Serum protein electrophoresis

Serum protein electrophoresis was performed on day 0 prior to and on days 44, 56/58 and 69 post *M. haemofelis* exposure. A semi-automated agarose gel electrophoresis system (Hydragel-Hydrasis, Sebia PN 4100, Issy-les Moulineaux, France) was used according to the manufacturer’s instructions (Hydragel 7 Protein kit, Sebia PN 4100). The reference ranges were determined for routine purposes using identical methods and serum samples from 59 clinically healthy cats and are stated as 5% and 95% quantiles.

### Quantification of bacterial loads

Total nucleic acids (TNA) were extracted from 100 μL of EDTA-anticoagulated blood using the MagNA Pure LC Total Nucleic Acid Isolation Kit (Roche Diagnostic) as previously described [2]. The saliva and rectal swabs were incubated in PBS at 40 °C for 10 min prior to TNA extraction as previously described [25]. TNA was extracted from 100 μL of urine using the MagNA Pure LC DNA Isolation Kit III (Bacteria, Fungi) (Roche Diagnostic). As pre-isolation steps, Bacterial lysis Buffer and Proteinase K were added, and the mixture was incubated at 65 °C for 10 min. The subsequent procedure was performed according to the manufacturer’s instructions.

TNA was eluted into 100 μL of elution buffer and stored at −20 °C until further use. During all nucleic acid extractions, negative controls of 100 μL of PBS were concurrently prepared with each batch of samples to monitor cross-contamination.

All TNA samples were tested using TaqMan® real-time qPCR for the presence and quantity of *M. haemofelis* and “*Cand.* M. turicensis” on an ABI PRISM 7700/7500 Sequence Detection System (Applied Biosystems, Rotkreuz, Switzerland) and a 10-fold serial dilution of plasmid standards as previously described [2,14]. For all PCR reactions, positive (plasmid standard) and negative controls (nuclease free water) were included in each assay.

### Serology

Antibodies to *M. haemofelis* and “*Cand.* M. turicensis” were measured using an ELISA based on recombinant *M. haemofelis* DnaK protein as previously described [8] and a serum dilution of 1:500. The signal-to-noise ratio was calculated by dividing the post- by the pre-infection absorbance values for each individual cat as previously described [8]. An ELISA signal ratio of ≥1.5 was considered serologically positive [8]. Samples were collected up to 371 days post *M. haemofelis* exposure and included in the measurements.

### Quantification of cytokine transcription levels

Blood samples for cytokine transcriptional analysis were collected on days 0, 2, 6, 9, 13, 20, 27, 34 and 48 post *M. haemofelis* exposure. One hundred microliters of EDTA-anticoagulated blood was collected and immediately mixed with 300 μL of mRNA lysis buffer (mRNA Isolation Kit I, Roche Diagnostics). The samples were stored at −80 °C within 1 h of collection. mRNA was purified using the mRNA Isolation Kit I and a MagNA Pure LC instrument (Roche Diagnostics) according to the manufacturer’s instructions. mRNA was eluted into 25 μL of elution buffer and stored at −80 °C until further use. First-strand cDNA was synthesized using the High Capacity cDNA Reverse Transcription Kit (Applied Biosystems) according to the manufacturer’s instructions. For each mRNA sample, the cDNA was synthesized in duplicate and pooled. The cDNA samples were stored at −20 °C until use.

TaqMan® real-time PCR assays were performed for the relative quantification of feline interferon-γ (IFN-γ), interleukin (IL) 4, 6, 10 and 12 and tumor necrosis factor-α (TNF-α) using primers and probes previously described [26–28]. For IL-5, the primers fIL-5_121f 5’-TGAATAGGCTGGTGGCAGAGA-3’, fIL-5_194r: 5’-CAGGTTCCCGTCGCCTATC-3’ and the probe fIL-5_143p: 5’-FAM-CTTGGCACTGCTCTCCACTCATCGAACT-TAMRA-3’ were designed; the reverse primer anneals over an exon-exon junction. The IL-5 reaction contained 0.9 μM forward and reverse primers and 0.25 μM probe. All of the cytokine assays were run using TaqMan® Fast Universal PCR Master Mix (2×) with an initial denaturation at 95 °C for 20 s followed by 45 cycles 95 °C for 3 s and 60 °C for 30 s. The transcription levels of the V-abl Abelson murine leukemia viral oncogene homolog (ABL) and zeta polypeptide (YWHAZ) genes served as references for normalization as described [29]. These two genes were found to be best for quantitative analysis in feline blood samples [29]. PCR assays were performed using a Rotor-Gene 6000 real-time rotary analyzer (Corbett, Mortlake, Australia). The calculation of mRNA expression levels from the Ct-values and the efficiencies of the cytokine and reference gene assays was performed using GeNorm version 3.5 [30]. Briefly, the cytokine transcription levels were normalized to the transcription levels of ABL and YWHAZ at each time point and used to calculate the relative transcription levels compared to day 0.

### Flow cytometry analysis of lymphocyte subsets

EDTA-anticoagulated blood samples for flow cytometry were collected on day 0 prior to *M. haemofelis* exposure and on days 1, 2, 6, 9, 20, 27, 41, 57, 62, 76 and 83 after *M. haemofelis* exposure and processed as previously described [11]. The following antibody combinations were used: 1) a fluorescein isothiocyanate (FITC)-conjugated mouse anti-feline CD5 antibody (f43, Southern Biotech, BioConcept, Allschwil, Switzerland) recognizing T lymphocytes [31] and an unconjugated mouse anti-feline MHCII antibody (H34A, VMRD, Qiagen, Leipzig, Germany); 2) an R-phycoerythrin (RPE)-conjugated mouse anti-feline CD4 antibody (Vpg34, AbD Serotec, Puchheim, Germany) and a fluorescein isothiocyanate (FITC)-conjugated mouse anti-feline CD25 antibody (kindly provided by M.B. Tompkins and G. Dean); 3) an unconjugated mouse anti-feline CD8 antibody (FT2, Southern Biotech) and a peridinin chlorophyll-a protein (PerCP)-conjugated rat anti-mouse CD45R/B220 antibody (RA3-6B2, BD Bioscience, Allschwil, Switzerland). Allophycocyanin (APC)-conjugated mouse IgG1 anti-CD8 antibody (BD Bioscience) and an RPE-goat anti-mouse IgG2b MHCII antibody (Southern Biotech) were used as secondary antibodies. An aliquot of blood from each cat was left unstained as a negative control. Flow cytometry was performed using the BD FACSCalibur™ platform (BD Bioscience) and CellQuestPro™ software. Forward and reverse scatter gates were set for the lymphocyte population, and up to 10 000 events were acquired for each sample. Each lymphocyte subset was calculated as the product of the absolute lymphocyte number (determined by hematology) and the subset percentage (determined by flow cytometry) as previously described [32].

### Statistics

The number of cats per experimental group was five; it was kept to a minimum for animal welfare reasons. At the same time, five animals per group are necessary to potentially reach statistical significance between the groups; even if one cat or the results of one cat per group are lost for unforeseeable reasons. Statistical analyses were performed using Graph-Pad Prism Version 3.0 (GraphPad Software, San Diego, CA, USA) and nonparametric tests. Parameters were compared between two groups using the Mann–Whitney U-test (p_MWU_). The Wilcoxon signed-rank test (p_W_) was used to compare the serum protein electrophoresis results from the same cat at two different time points. Friedman’s test (p_F_) followed by Dunn’s post test was used to analyze the parameters over time when more than two time points were considered. Multiplicity adjusted p-values for each comparison in a family of comparisons were computed. For the correlation analyses, the Spearman rank correlation test was used (p_S_), and the correlation (r) coefficient is given with the confidence interval (CI). *P*-values < 0.05 were considered significant.

## Results

### No cross-protection against *M. haemofelis* and earlier onset of infection in “*Cand.* M. turicensis”-recovered cats

At the start of the study, the blood from all ten cats was PCR-negative for *M. haemofelis* and “*Cand.* M. turicensis”. However, all cats became *M. haemofelis* PCR-positive after subcutaneous *M. haemofelis* exposure, and no cross-protection against *M. haemofelis* was observed in cats previously infected with “*Cand.* M. turicensis”. “*Cand.* M. turicensis” was not detectable by qPCR analysis of the blood samples from any of the cats. The cats in group A became *M. haemofelis* PCR-positive between days 9 and 16 after exposure, while the naïve control cats in group B became PCR-positive between days 20 and 34 (Figure 1). Thus, cats that had experienced “*Cand.* M. turicensis” infection became *M. haemofelis* PCR-positive significantly earlier than the naïve cats (p_MWU_ = 0.008). The maximum *M. haemofelis* blood loads were similar in both groups and reached >10^9^ copies/mL of blood. The cats in group A displayed fairly consistent high copy numbers (>10^7^ copies/mL) starting between days 21 and 31, which persisted until days 49 to 56 after *M. haemofelis* exposure. High bacterial loads (>10^7^ copies/mL) were observed later during infection and less consistently in cats in group B (starting between days 28 and 49 after exposure). Copy number fluctuations throughout the course of *M. haemofelis* infection were observed in all cats but were more pronounced in the cats in group B; the copy number fluctuations were particularly marked in cat ZKA2 in group B (up to a four-log fluctuation within four days; Figure 1G).

### Clinical signs of hemoplasmosis in naïve and “*Cand.* M. turicensis”-recovered cats

Two cats in group A (FIA1, FIA2) and three cats in group B (KCY2, ZKA2, JCT2) exhibited transient clinical signs of hemoplasmosis within the first nine weeks after subcutaneous exposure with *M. haemofelis*. The most severe outcome was observed in cat JCT2 in group B (Figure 1I); this cat was the last cat to become PCR-positive in the present study. The severe clinical signs, which necessitated antibiotic treatment, were described previously [19]; briefly, the cat had an enlarged abdomen and exhibited pale mucous membranes, apathy, anorexia and increased salivation. The other four cats (cats FIA1, FIA2, KCY2 and ZKA2) were depressed and exhibited pale mucous membranes and a reduced appetite over a period of a few days, but no treatment was required. No significant deviations in body temperature or body weight were observed in any cat during the course of the experiment.

### Early development of macrocytic hypochromic anemia after *M. haemofelis* exposure in “*Cand.* M. turicensis”-recovered cats

All five cats in group A and four of five cats in group B developed anemia (hematocrit <33%) in the present study (Figure 1). The cats in group A that previously had undergone “*Cand.* M. turicensis” infection displayed onset of anemia at 16 to 27 days post *M. haemofelis* exposure (6 to 13 days after PCR-positivity at times of maximal bacteremia in these cats), although one cat (FHT1) was previously slightly and transiently anemic with a minimum hematocrit of 30 on day 1 to 16 after exposure (Figure 1C). Interestingly, in the four of five naïve cats in group B that developed anemia, the onset of anemia occurred significantly later than in the cats in group A (34 to 62 days post *M. haemofelis* exposure; p_MWU_ = 0.0159). The lowest single hematocrit value among all cats was 13% (Figure 1I).

The cats in groups A and B exhibited significant differences over time in hematocrit (Additional file 2A; marked by an asterisk) and RBC (data not shown). The decreased red blood cell values coincided with maximal bacteremia. Overall, the hematocrit was significantly correlated with the *M. haemofelis* load (p_S_ < 0.0001; r = −0.46, CI −0.55 to −0.36).

The cats in group A also displayed significant differences over time in erythrocyte volume (MCV; Additional file 2B) and the hemoglobin concentration of the erythrocytes (MCHC; Additional file 2C). The altered erythrocyte indices (increased MCV; decreased MCHC) coincided with maximal bacteremia and anemia and were indicative of macrocytic hypochromic anemia. The erythrocyte indices returned to pre-exposure values more slowly than the hematocrit (data not shown). The cats in group B exhibited increased MCV and decreased MCHC similar to the cats in group A but at slightly later time points (Additional files 2E and F). None of the cats tested positive in Coombs’ test at the time point tested (late after infection; >300 days after *M. haemofelis* exposure).

### Decreased leukocytes, lymphocytes and eosinophils and increased monocytes in “*Cand.* M. turicensis”-recovered cats at times of maximal *M. haemofelis* bacteremia

**Figure 2:**
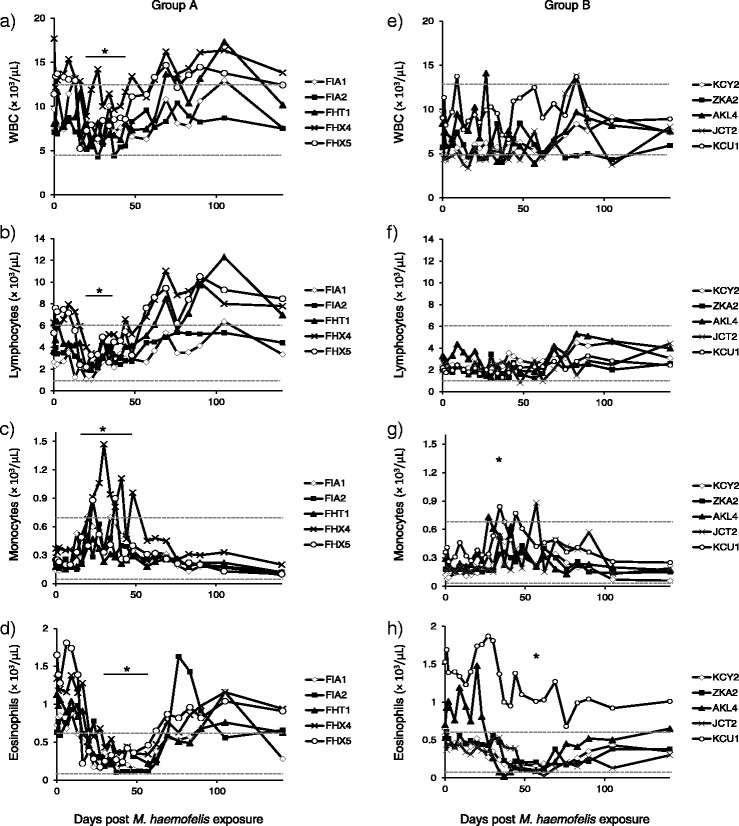
**Total leukocyte and leukocyte subset cell counts after** ***M. haemofelis*** **exposure in ten SPF cats.** The five cats in group A had undergone previous “*Cand.* M. turicensis” infection **(A**-**D)** and the five cats in group B were naïve control cats **(E**-**H)**. The *M. haemofelis* exposure took place on day 0. WBC **(A**, **E)**, lymphocytes **(B**, **F)**, monocytes **(C**, **G)** and eosinophils **(D**, **H)**. Significant decreases and increases over time are indicated with an asterisk, and durations spanning more than one time point are indicated as a solid black line (p_F_ < 0.05 and p_D_ < 0.05, unless indicated otherwise). In detail, significant differences over time were observed in WBC counts in the cats in group A with decreased WBC counts on days 20, 23, 30, 37 and 41 compared with days 65 and 109 post exposure (**A**). Significant differences over time were observed in lymphocyte counts for both groups with decreased lymphocyte counts on days 20, 23, 27 and 37 compared with days 62, 19, 83, 90, 105 and 141 post exposure in cats in group A **(B)** **(**no significance in the post test for group B; **F)**. A significant increase in monocyte counts with increased values on days 20 to 48 compared with days 141, 190, 232, 272 and 371 post exposure **(C)** and a decrease in eosinophil counts with decreased values on days 30, 37, 41, 48 and 57 compared with day 9 and day 286 after *M. haemofelis* exposure were observed in the cats in group A **(D)**. In group B an increase in monocytes with increased values on day 34 compared with days 105, 141, 190 and 371 post exposure **(G)** and a decrease in eosinophils were found decrease on day 57 compared with day 190 post exposure **(H)**. Upper and lower reference values are indicated as a dotted line.

The white blood cell (WBC) counts were not significantly different between groups A and B at day 0. Significant differences over time were observed in WBC counts in the cats in group A but not in the cats in group B (Figures 2A and E; low WBC counts in A marked with an asterisk). However, all four anemic cats in group B exhibited leukopenia at several time points throughout the study. Neutropenia was observed in several cats in both groups throughout the experiment but occurred most frequently and more severely in cat JCT2, which had to be treated due to severe clinical outcomes (minimal neutrophil count: 1.0 × 10^3^/μL on day 105). Remarkably, the cats in group A had higher lymphocyte counts than the naïve cats in group B at the start of the experiment (days 0 to 6 and 9; p_MWU_ = 0.0159; Figures 2B and F). Significant differences over time in lymphocyte counts were observed in group A (Figure 2B; decrease at maximal bacteremia, marked with an asterisk). Due to this decrease in lymphocyte counts in group A, there was no difference between the two groups from days 16 to 41 post exposure; thereafter, the cats in group A had higher lymphocyte counts than the cats in group B. In addition and parallel to the decrease in WBC and lymphocyte counts, a significant increase in monocyte counts (Figure 2C) and a decrease in eosinophil counts (Figure 2D) were observed in the cats in group A. In group B similar alterations but less pronounced were observed; these included increased monocytes (Figure 2G) and decreased eosinophils (Figure 2H).

### Early increase in T cells and decrease in several lymphocyte subsets at times of high bacteremia in *M. haemofelis*-exposed “*Cand.* M. turicensis”-recovered cats

**Figure 3:**
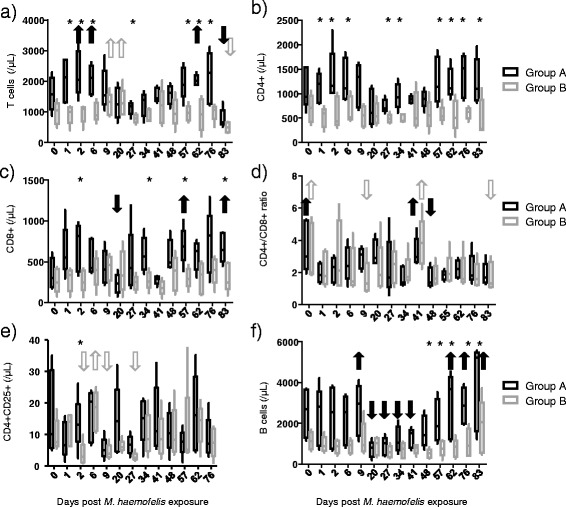
**Lymphocyte subsets after** ***M. haemofelis*** **exposure in the ten SPF cats.** The five cats in group A had undergone previous “*Cand.* M. turicensis” infection and the five cats in group B were naïve control cats. The *M. haemofelis* exposure took place on day 0. **A)** T cells (CD5 + MHCII+ ) counts, **B)** CD4+ T cell counts, **C)** CD8+ T cell counts, **D)** CD4+/CD8+ ratio, **E)** CD4+CD25+ activated T cells counts and **F)** B cell (CD45R/B220+) counts. No significant differences between groups A and B were observed on day 0; significant differences between the two groups observed thereafter are indicated with an asterisk (p_MWU_ < 0.05). Significant differences over time (p_F_ < 0.05 and p_D_ < 0.05) and the corresponding significantly decreased or increased values are indicated with an arrow (black solid for group A and gray open for group B). In detail, increased T cell counts were observed in group A on days 2, 6 and 62 compared with day 83 and in group B on day 9 and 20 compared with day 83 **(A)**. Decreased CD8+ T lymphocyte counts were observed in group A on day 20 compared with days 57 and 83 **(C)**. In the CD4+/CD8+ ratio, in group A, significantly lower values were observed on day 48 compared to days 0 and 41, whereas in group B, higher counts were observed on day 0 compared to days 9 and 83 and on and 41 compared to day 9 **(D)**. CD4+CD25+ cell counts varied significantly over time in cats in group B, with lower values on days 2, 9 and 27 compared with day 6 **(E)**. B cells varied significantly over time in cats in group A, with transiently decreased values on days 20, 27, 34 and 41 compared with days 9, 62, 76 and 83 **(F)**. No values were available for CD4+CD25+ on day 83.

Although the cats in group A had higher total lymphocyte counts on day 0, no significant differences in lymphocyte subsets were observed at that time point (Figure 3). However, the cats in group A had significantly higher T cell counts, CD4+ and CD8+ T lymphocyte counts, activated CD4+ T cell (CD4+CD25+) and B cell counts than the cats in group B at several time points throughout the study: T cells, CD4+ and CD8+ cells were higher in group A than in group B already within days after *M. haemofelis* exposure, while B cells were significantly higher in group A compared to group B later during the course of infection after maximal bacteremia (Figure 3A, B, C and E; all differences marked by asterisks). Significant differences over time were observed in T cell counts in both groups with increased values in group A within days after *M. haemofelis* exposure (Figure 3A; marked by arrows). In addition, cats in group B exhibited significant differences over time in activated CD4+ T cell (CD4+CD25+) counts also within days after *M. haemofelis* exposure (Figure 3E; marked by arrows). Significantly decreased CD8+ T lymphocyte counts were observed in group A at the onset of maximal bacteremia (day 20) compared with later time points (Figure 3C; marked by arrows), while B cells were decreased at times of maximal bacteremia in group A (days 20 to 41) and rebounded thereafter (Figure 3F; marked by arrows). Finally, the CD4+/CD8+ ratio significantly fluctuated over time, but no clear pattern was recognized (Figure 3D).

### Serum proteome in “*Cand.* M. turicensis”-recovered cats: polyclonal hypergammaglobulinemia at maximal bacteremia

**Figure 4:**
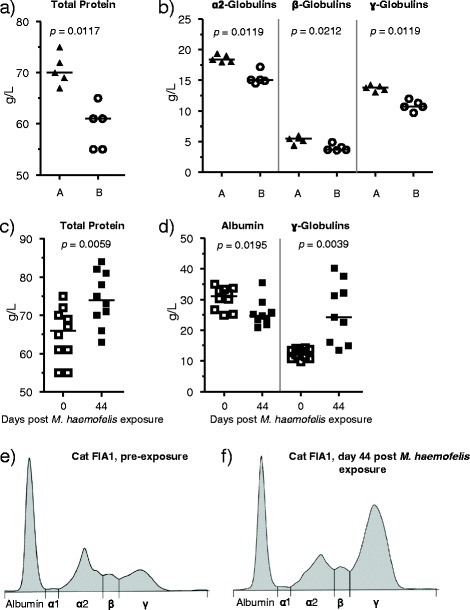
**Serum protein electrophoresis results.** **A** and **B**: pre-exposure values of total protein concentrations **(A)** and globulins **(B)** in the cats of groups **A** (black triangles) and **B** (open circles). **C** and **D**: comparison of the pre-exposure values (day 0; open squares) to those obtained on day 44 post *M. haemofelis* exposure (black squares). In **A** through **D**, the horizontal line indicates the median. *P*-values (**A** and **B**: p_MWU_, **C** and **D**: p_W_) are indicated in the figure. **E** and **F**: representative results for serum protein electrophoresis (of cat FIA1, group A) before **(E)** and at 44 days post *M. haemofelis* exposure **(F)**. Albumin concentrations were significantly lower and ɣ-globulin concentrations were significantly higher on day 44 after *M. haemofelis* exposure compared with the pre-exposure values (**D**; p_W_ < 0.05). The electrophoretogram conducted with a sample collected on day 44 **(F)** illustrates polyclonal hypergammaglobulinemia with a ɣ-globulin concentration of 40.2 g/L (reference range: 5.7-16.0 g/L).

When comparing the two groups, the cats in group A had significantly higher protein, albumin and globulin concentrations than the cats in group B prior to *M. haemofelis* exposure (day 0, p_MWU_ < 0.05; Figure 4A and Additional files 3A to C). The cats in group A exhibited a significant increase in total serum protein and globulin and a decrease in albumin particularly at 3 to 8 weeks after *M. haemofelis* exposure, consistent with maximal bacteremia in these cats (Additional files 3A to C). The cats in group B exhibited tendencies that were similar but less pronounced than those of group A (Additional files 3F to H). Globulin concentrations were significantly higher in group A than group B on days 23, 30 and 37, and the albumin concentration was lower on day 37 post exposure (p_MWU_ < 0.05; Additional file 3).

Serum protein electrophoresis prior to *M. haemofelis* exposure revealed that the cats in group A had significantly higher ɑ2-, β- and ɣ-globulin values than the cats in group B (p_MWU_ < 0.05; Figure 4B). Significant differences over time were observed when all ten cats were analyzed in combination (Figure 4C and D). Albumin concentrations were significantly lower and ɣ-globulin concentrations significantly higher at the tested time points after *M. haemofelis* exposure compared to pre-exposure values (Figure 4D; Additional files 3B and G). Nine of the ten cats had ɣ-globulin concentrations above the reference range (>16 g/L) at days 44, 56/58 or 69 (Figure 4D and data not shown). The albumin to globulin ratio was significantly decreased at all three tested time points after *M. haemofelis* exposure compared with pre-exposure values (p_W_ ≤ 0.0137; data not shown). Moreover, on day 44 after *M. haemofelis* exposure, the β-globulin fraction was significantly higher compared with the pre-exposure values (P_W_ ≤ 0.0091; data not shown). The electrophoretogram on day 44 post exposure indicated polyclonal hypergammaglobulinemia (Figure 4F).

### Increases or decreases in other clinical chemistry parameters at maximal *M. haemofelis* bacteremia

The cats in group A, and in group B albeit less pronounced, exhibited significant differences over time in additional clinical chemistry parameters throughout the experiment. Mostly 3 to 8 weeks after *M. haemofelis* exposure, consistent with maximal bacteremia, the following deviations were observed: decreased cholesterol (Additional files 3D and I), creatinine (Additional files 3E and K), alkaline phosphatase (Additional files 4A and D), sodium (Additional files 4C and F) and increased bilirubin (Additional files 4B and E). A significant inverse correlation was observed between the bilirubin concentration and the hematocrit values (p_S_ < 0.0001; r = −0.30, CI −0.45 to −0.15).

### Increase in anti-DnaK antibodies in “*Cand.* M. turicensis”-recovered cats after *M. haemofelis* exposure

The cats in group A that had previously experienced “*Cand.* M. turicensis” infection were serologically positive at the beginning of the study (Additional file 1A). These cats displayed a remarkable increase in antibody levels on days 13 to 23 post exposure (P_W_ = 0.0625) and had significantly higher antibody levels than the cats in group B from day 0 to 41 after exposure (p_MWU_ ≤ 0.0317; Additional file 1). The five naïve cats in group B were seronegative at the start of the study (ELISA signal-to-noise ratio <1.5; Additional file 1B). The cats seroconverted seven to ten days after the blood samples were identified as PCR-positive and 30 to 37 days after exposure, with the exception of cat JCT2, which seroconverted 55 days after *M. haemofelis* exposure. For JCT2, seroconversion occurred 21 days after the cat had been transiently identified as PCR-positive and ten days after the onset of persistent PCR-positivity. The cats in group A achieved maximum antibody production significantly earlier during acute infection (20 to 30 days post exposure) than the cats in group B (37 to 62 days post exposure; p_MWU_ = 0.0079), and the maximum antibody levels during acute infection were significantly higher in group A (19.0 – 30.3) than in group B (10.7 – 17.6; p_MWU_ = 0.0079). The antibody levels were significantly correlated with the *M. haemofelis* blood load (p_S_ < 0.0001; r = 0.57, CI 0.48 to 0.65).

### Cytokine transcription levels: predominance of the Th1 response at maximal *M. haemofelis* bacteremia

**Figure 5:**
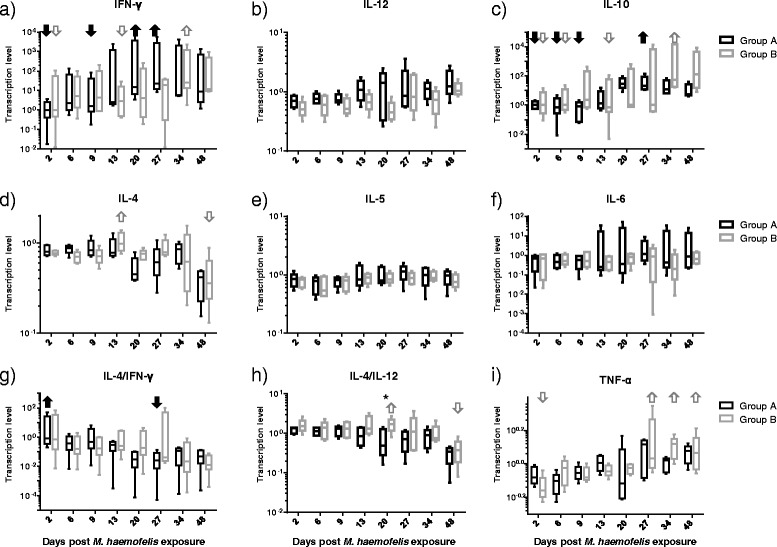
**Transcription levels of cytokines after** ***M. haemofelis*** **exposure in the ten SPF cats.** The five cats in group A had undergone previous “*Cand.* M. turicensis” infection and the five cats in group B were naïve control cats. The *M. haemofelis* exposure took place on day 0. **A)** IFN-γ, **B)** IL-12, **C)** IL-10, **D)** IL-4, **E)** IL-5, **F)** IL-6, **G)** IL4/IFN-γ ratio, **H)** IL-4/IL-12 ratio and **I)** TNF-α. Y-axis: relative transcription levels (log scale). Significant differences over time (p_F_ < 0.05 and p_D_ < 0.05) and the corresponding significantly decreased or increased values are indicated with an arrow (black solid for group A and gray open for group B). In detail, IFN-γ mRNA levels varied over time, with higher values on days 20 and 27 compared with days 2 and 9 in group A and on day 34 compared with days 2 and 13 in group B **(A)**. IL-10 mRNA levels increased throughout the study, with higher levels on day 27 compared with days 2, 6, and 9 in group A and higher levels on day 34 compared with days 2, 6 and 13 in group B **(C)**. IL-4 levels varied over time in both groups with lower IL-4 levels on day 48 compared with day 13 in group B (post test not significant for group A; **D)**. The IL-4/IFN-γ ratio varied in both groups over time, with a decreased in group A from day 2 to 27 (post test not significant for group B; **G)**. The IL-4/IL-12 ratio varied over time in both groups, with a decrease from days 20 to 48 in group B, and lower levels in group A compared with group B on day 20 **(H)**. TNF-α mRNA levels varied over time in both groups, with higher levels observed on days 27, 34 and 48 compared with day 2 in group B (post test not significant for group A; **I)**.

Similar patterns of cytokine transcription levels were observed in groups A and B, with a tendency of alterations occurring later in group B than in group A (Figure 5). IFN-γ (Th1) and IL-10 (anti-inflammatory) mRNA levels varied significantly over time in both groups with higher values at times of maximal bacteremia compared to values early after *M. haemofelis* exposure (Figures 5A and C; indicated by arrows). The increase in mean IFN-γ transcription levels was more pronounced in group A (day 2 to day 20: ~1100-fold increase) than in group B (day 2 to 34: ~20-fold increase), while the increase in IL-10 mean transcription levels was more pronounced in group B (day 2 to day 34: ~1700-fold increase) than in group A (day 2 to 27: ~50-fold increase). Moreover, a significant decrease in IL-4 (Th2) levels was observed in group B (Figure 5D). A significant decrease over time of the IL-4/IFN-γ ratio (Th2/Th1) particularly in group A (day 2 to day 27 post exposure: mean decrease ~250-fold) and of the IL-4/IL-12 ratio (Th2/Th1) ratio particularly in group B were observed (Figure 5G and H). In contrast, TNF-α mRNA levels significantly increased over time in both groups (Figure 5I). No significant differences over time were observed in IL-5, IL-6 and IL-12 transcription levels (Figures 5E, F and B). No significant differences were observed between groups A and B except for a difference in the IL-4/IL-12 at day 20 (Figure 5I; marked by an asterisk).

### Detection of low-level *M. haemofelis* shedding in saliva, feces and urine samples

Saliva and rectal swabs were collected at the time points indicated in Figure 1 and on days 141, 232 and 286 post *M. haemofelis* exposure. Some rectal swabs for all cats and some saliva swabs for eight of ten cats were PCR-positive for *M. haemofelis*. For two cats in group B (KCY2 and KCU1), all tested saliva swabs were negative (Figures 1F and J). The *M. haemofelis* loads in saliva and feces were between 200 to 780 copies/swab. Most of the positive results were obtained during high bacteremia. All saliva and rectal swabs collected on days 141, 232 and 286 post *M. haemofelis* exposure tested *M. haemofelis* PCR-negative (data not shown).

The urine samples for nine of ten cats tested PCR-positive for *M. haemofelis*; cat FIA2 in group A was negative on all four occasions tested (Figure 1B). The *M. haemofelis* loads in the urine samples were 2 × 10^2^ to 1.4 × 10^5^ copies/mL of urine. The highest *M. haemofelis* load in urine was observed in a sample with high erythrocyte contamination in urinalysis. Shedding in urine was not strictly associated with high blood loads (no significant correlation between blood and urine loads was observed, p_S_ > 0.05) or blood contamination of the urine sample (data not shown).

### Long-term outcome of *M. haemofelis* infection: recovery in the absence of antibiotic treatment

Remarkably, five cats became PCR-negative for the detection of *M. haemofelis* in the blood within the observation period without antibiotic treatment: four cats in group A (cat FIA1, FIA2, FHX4 and FHX5) and one cat in group B (KCY2). The remaining six cats were still PCR-positive at the end of the study, 371 days after *M. haemofelis* exposure (data not shown).

## Discussion

In the present study, no cross-protection against *M. haemofelis* was observed in any of the five “*Cand.* M. turicensis”-recovered cats. By contrast, cats previously infected with “*Cand.* M. turicensis” infection exhibited faster onset of the infection and became PCR-positive and anemic significantly earlier than the naïve *M. haemofelis*-exposed control cats. Moreover, the cats previously infected with “*Cand.* M. turicensis” had persistently high *M. haemofelis* bacterial loads (≥10^7^ copies/mL) and higher total, CD4+, CD8+ and CD4+CD25+ T cells than naïve cats within days after *M. haemofelis* exposure. There are several possible explanations for the finding that “*Cand.* M. turicensis”-recovered cats became *M. haemofelis* PCR-positive significantly earlier than naïve cats. Pre-existing antibodies against hemoplasmas might have impacted the kinetics of *M. haemofelis* infection in cats that had recovered from “*Cand.* M. turicensis” infection, e.g., through antibody-dependent enhancement (ADE). ADE can increase the replication and distribution of pathogens, particularly viruses, e.g., feline corona virus [33]. However, ADE has also been suspected in bacterial infection [34] because the specific antibody response against *Streptococcus pneumoniae* enhances infection. It has been speculated that bacterial enzymes can modify specific IgA1 antibodies so that the binding of bacteria to the target cells is not inhibited but augmented, thereby facilitating the prolonged persistence of the bacteria at the mucosal surfaces of the respiratory tract [34,35]. Because cross-reactivity of antibodies to feline hemoplasmas, at least to the currently available recombinant *M. haemofelis* antigen DnaK, has been demonstrated [8,9], the occurrence of a similar phenomenon in “*Cand.* M. turicensis”-recovered cats with high antibody levels prior to *M. haemofelis* exposure cannot be excluded. Alternatively, chronically “*Cand.* M. turicensis”-infected cats may have become *M. haemofelis* PCR-positive earlier than naïve cats due to modulation or down-regulation of the immune system. The influence of a hemotropic mycoplasma infection on the host immune system has not yet been completely elucidated. For non-hemotropic mycoplasma infections, a multitude of mechanisms have been described that might modulate or down-regulate the cell-mediated and/or humoral immune response of the infected host [36].

None of these hypotheses, however, can adequately explain the observed enhancement after heterologous (“*Cand.* M. turicensis”-*M. haemofelis*) but not homologous challenge (“*Cand.* M. turicensis”-“*Cand. M. turicensis*” and *M. haemofelis*-*M. haemofelis*) [10,12,11]. Based on the immune response after hemoplasma infections, a Th1 response was suspected in primary *M. haemofelis* infection in naïve cats [12], while primary “*Cand.* M. turicensis” infection in naïve cats might be associated with an early Th1 response, with a subsequent switch to Th2 [11]. In cats that had recovered from “*Cand.* M. turicensis” infection, protection from reinfection was associated with an early and pronounced Th2 response [11]. While these results require further confirmation, the Th1/Th2 response may have at least partially contributed to the lack of cross-protection and the differences in the infection kinetics observed in the present study. If indeed “*Cand.* M. turicensis” infection leads to the stimulation of the humoral immune response (Th2), this effect might be counterproductive during subsequent *M. haemofelis* exposure, in which protection was observed in the absence of a Th2 response [12]. At the start of the study, in addition to the seropositivity for DnaK, “*Cand.* M. turicensis”-recovered cats had higher total lymphocyte counts, protein, albumin and ɑ2-, β- and ɣ-globulin concentrations than the naïve control cats, indicating immune system stimulation in “*Cand.* M. turicensis”-recovered cats. In particular, the increase in globulin levels may indicate a predominance of the humoral immune response in these cats. Thus, the “*Cand.* M. turicensis”-recovered cats, despite or even due to the observed seropositivity and the status of the immune system, may have been ill-prepared for subsequent *M. haemofelis* exposure. Consistently, within days after *M. haemofelis* exposure, “*Cand.* M. turicensis”-recovered cats exhibited higher total, CD4+, CD8+ and reactivated (CD4+CD25+) T-cell counts than the control cats.

Particularly in lymphocytes and lymphocyte subsets, differences were observed between the naïve cats and the “*Cand.* M. turicensis”-recovered cats in that “*Cand.* M. turicensis”-recovered cats but not naïve cats showed a transient decrease in lymphocyte subsets, especially in B cells, during maximal bacteremia. In a previous study, a transient decrease in lymphocytes during *M. haemofelis* infection was attributed to cell migration from the blood to the draining lymph nodes, where lymphocytes become activated and proliferative [12]. The increase in total protein concentrations suggests B cell activation during high bacteremia. This increase was paralleled by a decrease in albumin, possibly due to the role of albumin as a negative acute phase protein [37]. The increase in total protein at the time of maximal bacteremia is primarily attributable to the increased production of immunoglobulins, as demonstrated by the detection of polyclonal hypergammaglobulinemia in the serum protein electrophoresis of samples obtained from nine of ten cats. While the observed hypergammaglobulinemia coincided with the high specific antibodies to *M. haemofelis* determined by recombinant *M. haemofelis* DnaK antigen ELISA, a large proportion of the immunoglobulins were probably not hemoplasma-specific, reflecting polyclonal B cell activation. Mitogenicity and the activation of lymphocytes in a non-specific polyclonal manner has been well described for non-hemotropic mycoplasmas [36]. For *Mycoplasma suis*, a porcine hemoplasma, significant alterations to the RBC surfaces lead to the upregulation of naturally occurring B cells specific for self-antigens and the induction of auto-reactive IgM and IgG, which in turn leads to extra and intravascular hemolysis [38]. A previous study demonstrated that *M. haemofelis*-infected cats harbor antibodies directed against the erythrocyte surface (Coombs’ test positive) at the nadir of hematocrit values and at times of maximal bacteremia [24]. In the current study, Coombs’ test was negative, but this test was performed at a later time point during infection, when B cell counts and immunoglobulin values had returned to pre-infection values.

Several alterations in clinical chemistry parameters associated with high *M. haemofelis* blood loads were observed, such as decreased cholesterol. Alterations in lipid metabolism and decreased cholesterol levels have been reported in many infections, including viral, bacterial and parasitic infections [39,40]. The observed decrease in cholesterol concentration in the present study might reflect an increased need for cholesterol for membrane synthesis for either large numbers of RBCs destroyed during the *M. haemofelis* infection and/or the fast amplification of hemoplasmas. Mycoplasmas depend on cholesterol from the host for membrane synthesis and they incorporate large quantities of cholesterol into the membrane for improved membrane fluidity [36].

Moreover, a significant increase in bilirubin concentration was observed at some time points. As previously described [1], acute *M. haemofelis* infection may lead to hemolytic anemia, resulting in an increase in the bilirubin concentration. In the present study, the bilirubin concentration was directly associated with the bacterial loads, which may be supportive of a causal connection.

The cytokine response observed during *M. haemofelis* infection in the present study was predominantly of the Th1 type, consistent with previous results for naïve cats infected with *M. haemofelis* [12]. In the present study, naïve but particularly “*Cand.* M. turicensis”-recovered cats displayed a massive (~1100-fold) increase in IFN-γ transcription levels approximately coincident with the appearance of high bacterial loads. This effect was paralleled by a pronounced decrease in the IL-4/IFN-γ ratio (~250-fold). These alterations suggest the predominance of a Th1 response in both groups after the establishment of high bacterial loads in these cats, in contrast to the predominance of a Th2 response with high IL-4 transcription levels, a high IL-4/IL-12 ratio and an increase in eosinophils observed in “*Cand.* M. turicensis”-infected cats during high “*Cand.* M. turicensis” bacteremia [11]. Consistent with the absence of a Th2 response, no eosinophilia was observed in the present study; rather, a decrease in eosinophils was observed at times of maximal bacteremia. The Th1 response postulated in the *M. haemofelis*-infected cats in the present study might at first glance be contradicted by the major increase in IL-10 levels particularly in naïve cats (~1700-fold) also observed during high bacterial loads. An increase in IL-10 transcription levels was also observed in a previous study in naïve cats infected with *M. haemofelis* but not in cats protected from *M. haemofelis* re-infection [12]. IL-10 has multiple effects [41]: it inhibits Th1 cells and may be associated with B cell proliferation and antibody production. Thus, the high IL-10 transcription levels observed at times of high bacteremia would be consistent with the high serum protein, γ-globulin and anti-*M. haemofelis* DnaK antibody levels observed at the same time. However, IL-10 is also considered a potent anti-inflammatory cytokine that may limit the damage to the host due to exacerbated inflammation while prolonging pathogen clearance [41,42]. Therefore, it has been hypothesized that the high IL-10 levels observed during maximal bacteremia in *M. haemofelis*-infected cats in a previous study might limit the inflammatory response to *M. haemofelis* and potentially prevent the clearance of these organisms [12]. Whether such an anti-inflammatory effect is beneficial during bacterial infections and sepsis has been debated. It is unknown if high (anti-inflammatory) IL-10 levels observed in the present study are associated with the decreased leukocyte counts and neutropenia observed during high bacteremia; among other hematological alterations, leukocytosis and neutrophilia are characteristics of inflammation. A reduction in local and systemic inflammation in connection with IL-10 may reflect the inhibition of cell trafficking, particularly the recruitment of neutrophils to the injury site, via inhibition of the production of neutrophil chemoattractants [42].

When measuring antibodies to *M. haemofelis*, “*Cand.* M. turicensis”-recovered cats displayed an earlier and higher increase in anti-DnaK antibodies than naïve cats. By contrast, no increase in antibodies was observed in cats protected from homologous reinfection [12,11]; indeed, in cats that had undergone “*Cand.* M. turicensis” infection, a transient decrease in antibodies was observed upon homologous “*Cand.* M. turicensis” re-exposure, potentially reflecting the binding of antibodies to the inoculated antigens [10,11]. The levels of antibodies observed in the present study in response to *M. haemofelis* infection were higher than the level of antibodies reported in “*Cand.* M. turicensis” infection [10], consistent with the results of previous studies using a similar preparation of the same antigen [9]. This finding might reflect the higher blood loads obtained after *M. haemofelis* exposure compared with “*Cand.* M. turicensis” and the use of DnaK of *M. haemofelis* and not “*Cand.* M. turicensis” as the antigen for ELISA [8].

The shedding patterns of *M. haemofelis* were also investigated in the present study. This study is the first to report shedding of *M. haemofelis* DNA in saliva, feces and urine. Positive saliva and rectal swabs were obtained during high bacteremia, but only infrequently and not persistently. This finding might explain why previous studies were unable to detect *M. haemofelis* in saliva and salivary gland samples collected at only one time point or late during infection in only a few cats [22,43]. Moreover, the *M. haemofelis* loads observed in salivary swabs (a few hundred copies/swab) in the present study corresponded to those of “*Cand.* M. turicensis”-infected cats, as reported in previous studies [21,22]. Because “*Cand.* M. turicensis”-infected cats fairly consistently shed “*Cand.* M. turicensis” via saliva during high bacteremia [21,22] but the maximal blood loads were lower than in *M. haemofelis*-infected cats, factors other than blood load must play a role in whether feline hemoplasmas are shed via saliva. No *M. haemofelis* transmission studies using PCR-positive saliva or feces have been conducted. Transmission studies have been performed using “*Cand.* M. turicensis” PCR-positive saliva, but naïve cats did not demonstrate infection after oral uptake or subcutaneous inoculation of “*Cand.* M. turicensis” PCR-positive saliva [21]. In the present study, *M. haemofelis*-positive urine samples were also observed. Because the samples were collected through cystocentesis, we cannot completely exclude a contribution of erythrocyte contamination to the PCR-positive result. However, *M. haemofelis* PCR-positive results were also detected in urine samples in which no erythrocytes were detectable using urine test strips and sediment analyses. The low *M. haemofelis* copy numbers in the saliva, feces and urine samples of *M. haemofelis*-infected cats and the infrequent shedding, even during high bacteremia, suggest a minimal risk of transmission through mutual grooming and the sharing of feeding dishes or litter boxes; however, this should be confirmed in future studies.

In conclusion, in contrast to homologous challenge, no protection against heterologous *M. haemofelis* challenge was observed in cats that had recovered from a previous “*Cand.* M. turicensis” infection, indicating that “*Cand.* M. turicensis” is probably not a good candidate for an attenuated vaccine against the more pathogenic *M. haemofelis*. The differences in the immune reaction patterns (Th1 versus Th2) after “*Cand.* M. turicensis” and *M. haemofelis* infections were further corroborated. The study provides the first evidence of the presence of polyclonal hypergammaglobulinemia in cats infected with feline hemoplasmas. Moreover, recovered cats had higher lymphocyte counts. Finally, the study also suggests that a previous hemoplasma infection, even when the cat has ostensibly recovered, may influence subsequent infections and potentially lead to an enhancement phenomenon or differences in infection kinetics.

## Additional files

Additional file 1:
**Antibody response to DnaK after** ***M. haemofelis*** **exposure in ten SPF cats.** The five cats in group A had undergone previous “*Cand.* M. turicensis” infection (A) and the five cats in group B were naïve control cats (B). The *M. haemofelis* exposure took place on day 0. The antibody levels are presented as the signal-to-noise ratio determined by *M. haemofelis* rDnaK ELISA. A signal-to-noise ratio ≥1.5 (indicated by a dotted line) was defined as positive [8]. The cats in group A had significantly higher antibody levels than the cats in group B from days 0 to 41 (indicated by a line and an asterisk). The data from group B have been partially previously presented [19]. Additional file 2:
**Red cell parameters after** ***M. haemofelis*** **exposure in the ten SPF cats.** The five cats in group A had undergone previous “*Cand.* M. turicensis” infection (A-C) and the five cats in group B were naïve control cats (D-F). The *M. haemofelis* exposure took place on day 0. Hematocrit (A, D), mean corpuscular volume (MCV; B, E) and mean corpuscular hemoglobin concentration (MCHC, (C, F). Significant decreases and increases over time are indicated with an asterisk, and durations spanning more than one time point are indicated as a solid black line. The cats in group A exhibited significant differences over time in the hematocrit (p_F_ < 0.0001; decreased values on days 27, 30, 34, 37, 41 and 48 compared with days 0, 83, 90 and 141: p_D_ < 0.05; A) and RBC counts (p_F_ < 0.0001; decreased values on days 27 to 48 compared with pre-exposure and days 83, 90 and 141 after exposure: p_D_ < 0.05; data not shown). Similar alterations were observed in cats in group B (hematocrit: p_F_ ≤ 0.0005; decreased values on day 62 compared with days 141 and 280: p_D_ < 0.05, D; RBC: p_F_ < 0.0001; decreased values on day 69 and 105 compared with day 141: p_D_ < 0.05; data not shown). The cats in group A displayed also significant difference over time in MCV (p_F_ < 0.0001; increased values on days 27, 34, 37, 41, 48 and 57 compared with day 2 and days 190, 232 and 272 post exposure: p_D_ < 0.05; B) and the hemoglobin concentration of the erythrocytes (MCHC) (p_F_ < 0.0001; decreased values on days 27, 30 and 41 compared with day 2 and days 190, 232, 371 post exposure: p_D_ < 0.05; C). The cats in group B exhibited alterations similar to those in group A but at slightly later time points (MCV and MCHC: p_F_ < 0.0001; no significance in the post tests; E and F). Upper and lower reference values are indicated as a dotted line. Additional file 3:
**Selected clinical chemistry parameters after** ***M. haemofelis*** **exposure in the ten SPF cats I.** The five cats in group A had undergone previous “*Cand.* M. turicensis” infection (A-E) and the five cats in group B were naïve control cats (F-J). The *M. haemofelis* exposure took place on day 0. Total protein (A, F), albumin (B, G), globulin (C, H), cholesterol (D, I), and creatinine (E, K). The cats in group A had significantly higher protein, albumin and globulin concentrations at the beginning of the experiment than the cats in group B (p_MWU_ < 0.05; A to C and F to H). Significant increases and decreases over time are indicated with an asterisk, and durations spanning more than one time point are indicated as a solid black line. The cats in group A exhibited significant alterations over time in total serum protein (p_F_ < 0.0001; increased values on days 30 and 44 compared with days 16, 232 and 286: p_D_ < 0.05, A), albumin (p_F_ < 0.0001; decreased values on days 30, 37, 44 and 57 compared with days 0, 286 and 328: p_D_ < 0.005, B) and globulin (p_F_ < 0.0001; increased values on days 30, 37 and 44 compared with days 16, 232, 286 and 371: p_D_ < 0.005, C). The cats in group B exhibited similar but less pronounced alterations than the cats in group A in total protein (p_F_ < 0.0001; increased values on day 69 compared with days 0 and 23: p_D_ < 0.05; F), albumin (p_F_ < 0.0001; decreased values on day 57 compared with days 232, 328 and 371: p_D_ < 005; G) and globulin (p_F_ = 0.0015; increased values on day 69 compared with day 0: p_D_ < 0.005; H). Moreover, the cats in group A also exhibited significant alterations over time in: cholesterol (p_F_ < 0.0001; decreased values on days 30, 37, 44 and 57 compared with days 0, 190, 272, 286 and 328: p_D_ < 0.05; D) and creatinine (p_F_ < 0.0001; decreased values on days 30, 57 and 69 compared with days 141 and 328: p_D_ < 0.05; E). Similarly, the cats in group B exhibited differences over time in: cholesterol (p_F_ < 0.0001; decreased values on days 37, 44, 57 and 83 compared with days 9, 328 and 371: p_D_ < 0.05; I) and creatinine (p_F_ < 0.0001; decreased values on days 30, 57 and 69 compared with days 0 and 141: p_D_ < 0.05; K). Upper and lower reference values are indicated as a dotted line. Additional file 4:
**Selected clinical chemistry parameters after** ***M. haemofelis*** **exposure in the ten SPF cats II.** The five cats in group A had undergone previous “*Cand.* M. turicensis” infection (A-C) and the five cats in group B were naïve control cats (D-F). The *M. haemofelis* exposure took place on day 0. Alkaline phosphatase (A, C), bilirubin (B, E) and sodium (C,F). Significant increases and decreases over time are indicated with an asterisk, and durations spanning more than one time point are indicated as a solid black line. The cats in group A exhibited significant differences over time in: alkaline phosphatase (p_F_ < 0.0001; decreased values on days 23, 30, 37 and 44 compared with days 286, 328 and 371: p_D_ < 0.05; A), bilirubin (p_F_ = 0.0001; increased values on days 37 and 141 compared with day 328: p_D_ < 0.05; B) and sodium (p_F_ = 0.0001; decreased values on days 37, 57, and 69 compared with days 0, 328 and 371: p_D_ < 0.05; C). Similarly, the cats in group B exhibited significant alterations over time in: alkaline phosphatase (p_F_ = 0.0005; no significance in the post test; D), bilirubin (p_F_ = 0.0387; no significance in the post test; E) and sodium (p_F_ = 0.0001; decreased values on days 57 and 83 compared with days 328 and 371: p_D_ < 0.05; F). Bilirubin concentrations were higher in group A than in group B 30 days after *M. haemofelis* exposure (p_MWU_ < 0.05; B, E). Upper and lower reference values are indicated as a dotted line.
