# Supplementary material for: Lack of cross-protection against Mycoplasma haemofelis infection and signs of enhancement in “Candidatus Mycoplasma turicensis”-recovered cats
Source: Vet Res. 2015 Sep 24;46(1):104. doi: 10.1186/s13567-015-0240-x (PMC4581119; doi:10.1186/s13567-015-0240-x)
Supplement: Additional file 1: — Antibody response to DnaK after M. haemofelis exposure in ten SPF cats. The five cats in group A had undergone previous “Cand. M. turicensis” infection (A) and the five cats in group B were naïve control cats (B). The M. haemofelis exposure took place on day 0. The antibody levels are presented as the signal-to-noise ratio determined by M. haemofelis rDnaK ELISA. A signal-to-noise ratio ≥1.5 (indicated by a dotted line) was defined as positive [8]. The cats in group A had significantly higher antibody levels than the cats in group B from days 0 to 41 (indicated by a line and an asterisk). The data from group B have been partially previously presented [19]. [file 13567_2015_240_MOESM1_ESM.pptx]

## Slide 1
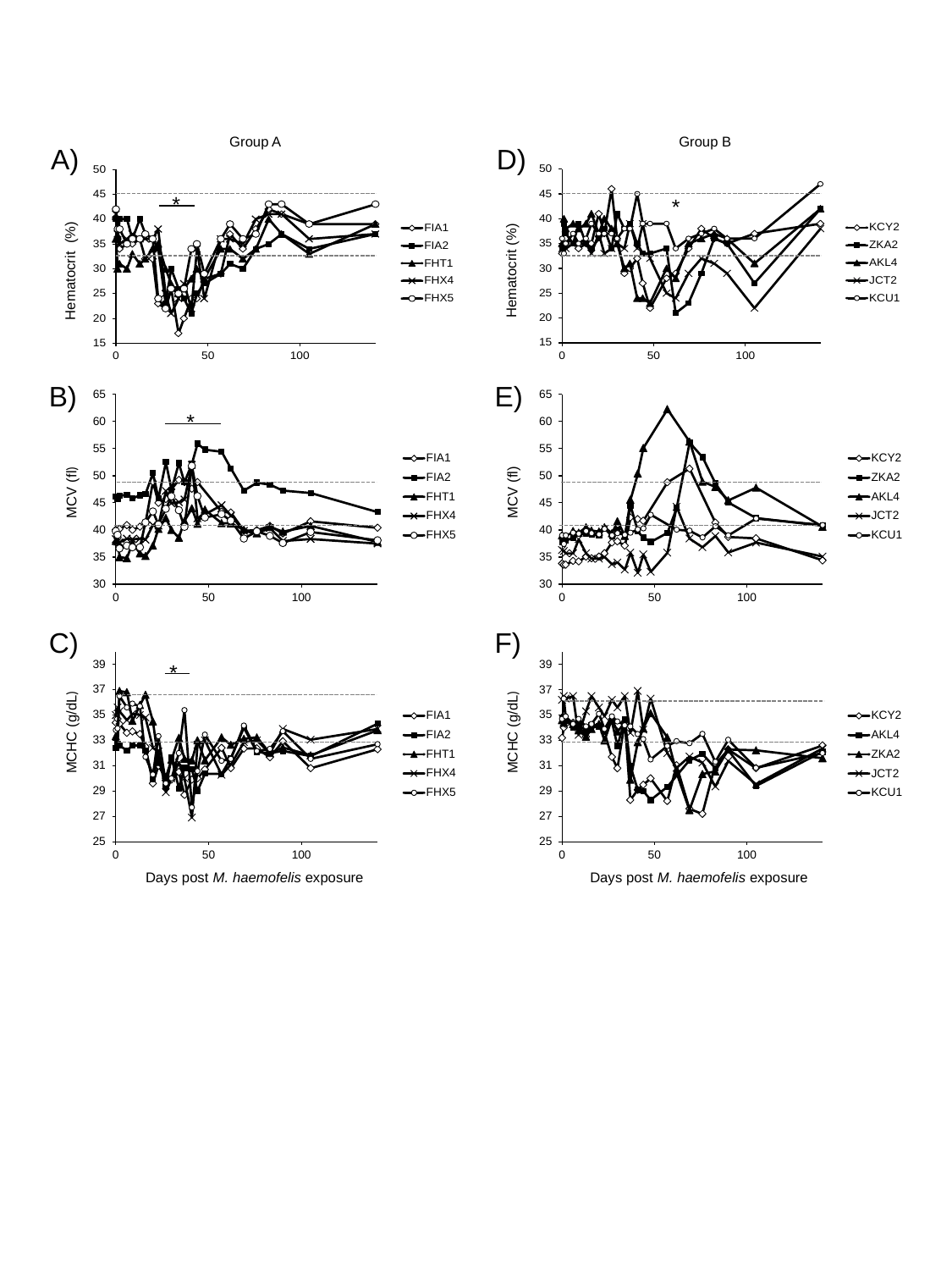

Group A
Group B
A)
D)
*
*
Hematocrit (%)
Hematocrit (%)
B)
E)
*
MCV (fl)
MCV (fl)
C)
F)
*
MCHC (g/dL)
MCHC (g/dL)
Days post M. haemofelis exposure
Days post M. haemofelis exposure
