# Supplementary material for: Lack of cross-protection against Mycoplasma haemofelis infection and signs of enhancement in “Candidatus Mycoplasma turicensis”-recovered cats
Source: Vet Res. 2015 Sep 24;46(1):104. doi: 10.1186/s13567-015-0240-x (PMC4581119; doi:10.1186/s13567-015-0240-x)
Supplement: Additional file 2: — Red cell parameters after M. haemofelis exposure in the ten SPF cats. The five cats in group A had undergone previous “Cand. M. turicensis” infection (A-C) and the five cats in group B were naïve control cats (D-F). The M. haemofelis exposure took place on day 0. Hematocrit (A, D), mean corpuscular volume (MCV; B, E) and mean corpuscular hemoglobin concentration (MCHC, (C, F). Significant decreases and increases over time are indicated with an asterisk, and durations spanning more than one time point are indicated as a solid black line. The cats in group A exhibited significant differences over time in the hematocrit (pF < 0.0001; decreased values on days 27, 30, 34, 37, 41 and 48 compared with days 0, 83, 90 and 141: pD < 0.05; A) and RBC counts (pF < 0.0001; decreased values on days 27 to 48 compared with pre-exposure and days 83, 90 and 141 after exposure: pD < 0.05; data not shown). Similar alterations were observed in cats in group B (hematocrit: pF ≤ 0.0005; decreased values on day 62 compared with days 141 and 280: pD < 0.05, D; RBC: pF < 0.0001; decreased values on day 69 and 105 compared with day 141: pD < 0.05; data not shown). The cats in group A displayed also significant difference over time in MCV (pF < 0.0001; increased values on days 27, 34, 37, 41, 48 and 57 compared with day 2 and days 190, 232 and 272 post exposure: pD < 0.05; B) and the hemoglobin concentration of the erythrocytes (MCHC) (pF < 0.0001; decreased values on days 27, 30 and 41 compared with day 2 and days 190, 232, 371 post exposure: pD < 0.05; C). The cats in group B exhibited alterations similar to those in group A but at slightly later time points (MCV and MCHC: pF < 0.0001; no significance in the post tests; E and F). Upper and lower reference values are indicated as a dotted line. [file 13567_2015_240_MOESM2_ESM.pptx]

## Slide 1
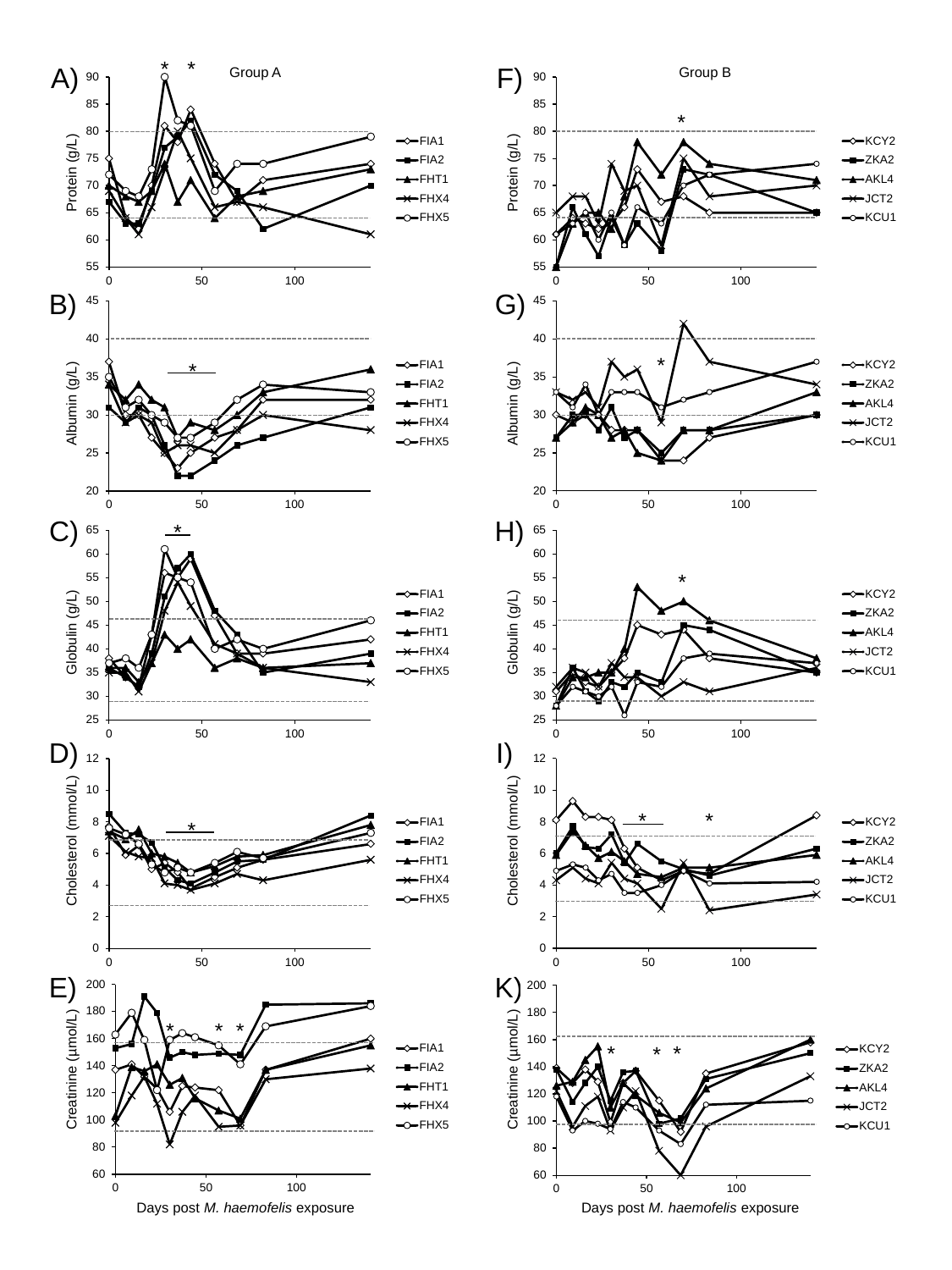

*
*
A)
F)
Group A
Group B
*
Protein (g/L)
Protein (g/L)
B)
G)
*
*
Albumin (g/L)
Albumin (g/L)
C)<
H)
*
*
Globulin (g/L)
Globulin (g/L)
D)
I)
*
*
*
Cholesterol (mmol/L)
Cholesterol (mmol/L)
E)
K)
*
*
*
*
*
*
Creatinine (µmol/L)
Creatinine (µmol/L)
Days post M. haemofelis exposure
Days post M. haemofelis exposure
