# Supplementary material for: Lack of cross-protection against Mycoplasma haemofelis infection and signs of enhancement in “Candidatus Mycoplasma turicensis”-recovered cats
Source: Vet Res. 2015 Sep 24;46(1):104. doi: 10.1186/s13567-015-0240-x (PMC4581119; doi:10.1186/s13567-015-0240-x)
Supplement: Additional file 3: — Selected clinical chemistry parameters after M. haemofelis exposure in the ten SPF cats I. The five cats in group A had undergone previous “Cand. M. turicensis” infection (A-E) and the five cats in group B were naïve control cats (F-J). The M. haemofelis exposure took place on day 0. Total protein (A, F), albumin (B, G), globulin (C, H), cholesterol (D, I), and creatinine (E, K). The cats in group A had significantly higher protein, albumin and globulin concentrations at the beginning of the experiment than the cats in group B (pMWU < 0.05; A to C and F to H). Significant increases and decreases over time are indicated with an asterisk, and durations spanning more than one time point are indicated as a solid black line. The cats in group A exhibited significant alterations over time in total serum protein (pF < 0.0001; increased values on days 30 and 44 compared with days 16, 232 and 286: pD < 0.05, A), albumin (pF < 0.0001; decreased values on days 30, 37, 44 and 57 compared with days 0, 286 and 328: pD < 0.005, B) and globulin (pF < 0.0001; increased values on days 30, 37 and 44 compared with days 16, 232, 286 and 371: pD < 0.005, C). The cats in group B exhibited similar but less pronounced alterations than the cats in group A in total protein (pF < 0.0001; increased values on day 69 compared with days 0 and 23: pD < 0.05; F), albumin (pF < 0.0001; decreased values on day 57 compared with days 232, 328 and 371: pD < 005; G) and globulin (pF = 0.0015; increased values on day 69 compared with day 0: pD < 0.005; H). Moreover, the cats in group A also exhibited significant alterations over time in: cholesterol (pF < 0.0001; decreased values on days 30, 37, 44 and 57 compared with days 0, 190, 272, 286 and 328: pD < 0.05; D) and creatinine (pF < 0.0001; decreased values on days 30, 57 and 69 compared with days 141 and 328: pD < 0.05; E). Similarly, the cats in group B exhibited differences over time in: cholesterol (pF < 0.0001; decreased values on days 37, 44, 57 and [file 13567_2015_240_MOESM3_ESM.pptx]

## Slide 1
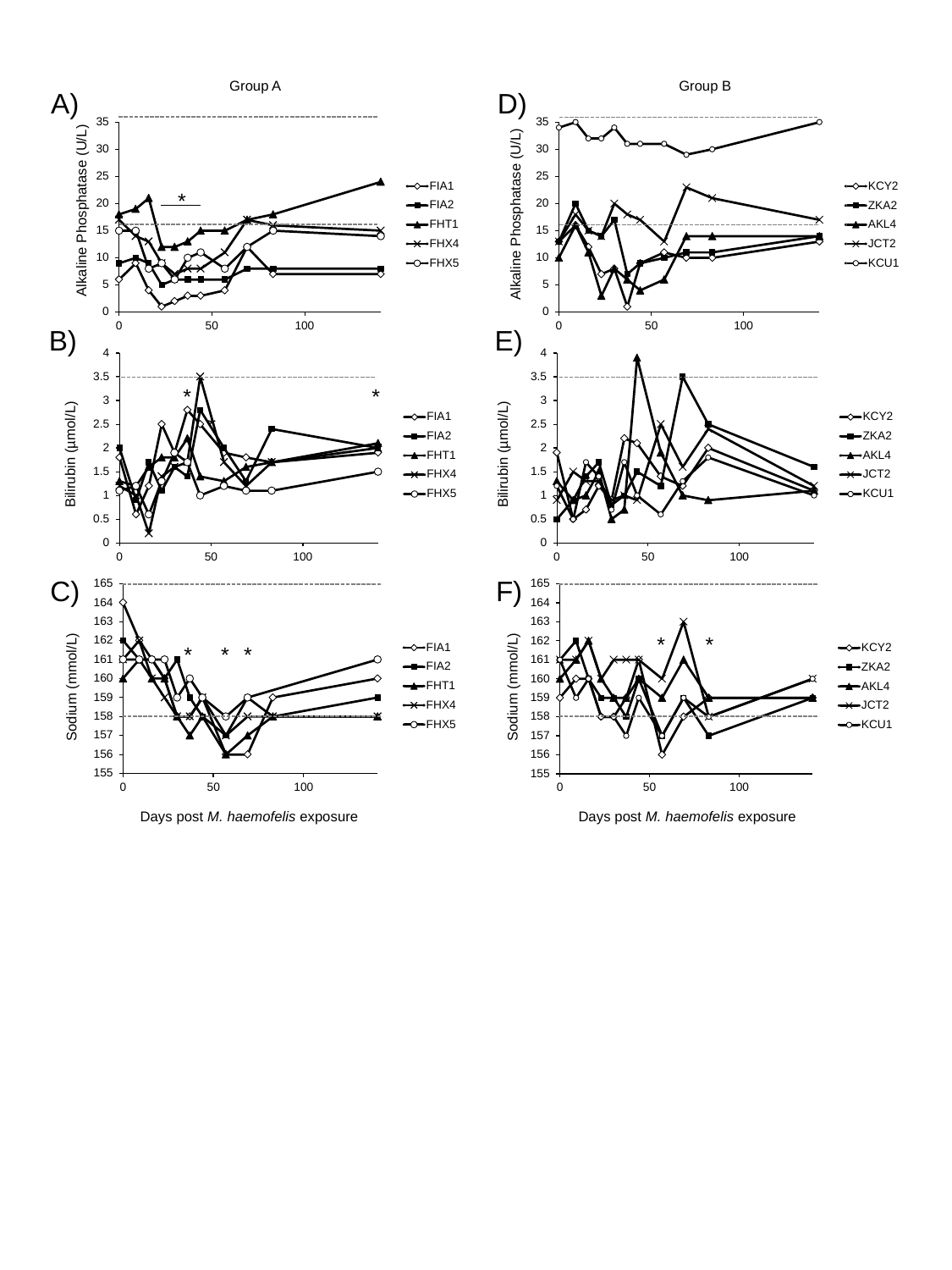

Group A
Group B
A)
D)
*
Alkaline Phosphatase (U/L)
Alkaline Phosphatase (U/L)
B)
E)
*
*
*
Bilirubin (µmol/L)
Bilirubin (µmol/L)
C)
F)
*
*
*
*
*
Sodium (mmol/L)
Sodium (mmol/L)
Days post M. haemofelis exposure
Days post M. haemofelis exposure
