# Supplementary material for: Lack of cross-protection against Mycoplasma haemofelis infection and signs of enhancement in “Candidatus Mycoplasma turicensis”-recovered cats
Source: Vet Res. 2015 Sep 24;46(1):104. doi: 10.1186/s13567-015-0240-x (PMC4581119; doi:10.1186/s13567-015-0240-x)
Supplement: Additional file 4: — Selected clinical chemistry parameters after M. haemofelis exposure in the ten SPF cats II. The five cats in group A had undergone previous “Cand. M. turicensis” infection (A-C) and the five cats in group B were naïve control cats (D-F). The M. haemofelis exposure took place on day 0. Alkaline phosphatase (A, C), bilirubin (B, E) and sodium (C,F). Significant increases and decreases over time are indicated with an asterisk, and durations spanning more than one time point are indicated as a solid black line. The cats in group A exhibited significant differences over time in: alkaline phosphatase (pF < 0.0001; decreased values on days 23, 30, 37 and 44 compared with days 286, 328 and 371: pD < 0.05; A), bilirubin (pF = 0.0001; increased values on days 37 and 141 compared with day 328: pD < 0.05; B) and sodium (pF = 0.0001; decreased values on days 37, 57, and 69 compared with days 0, 328 and 371: pD < 0.05; C). Similarly, the cats in group B exhibited significant alterations over time in: alkaline phosphatase (pF = 0.0005; no significance in the post test; D), bilirubin (pF = 0.0387; no significance in the post test; E) and sodium (pF = 0.0001; decreased values on days 57 and 83 compared with days 328 and 371: pD < 0.05; F). Bilirubin concentrations were higher in group A than in group B 30 days after M. haemofelis exposure (pMWU < 0.05; B, E). Upper and lower reference values are indicated as a dotted line. [file 13567_2015_240_MOESM4_ESM.pptx]

## Slide 1
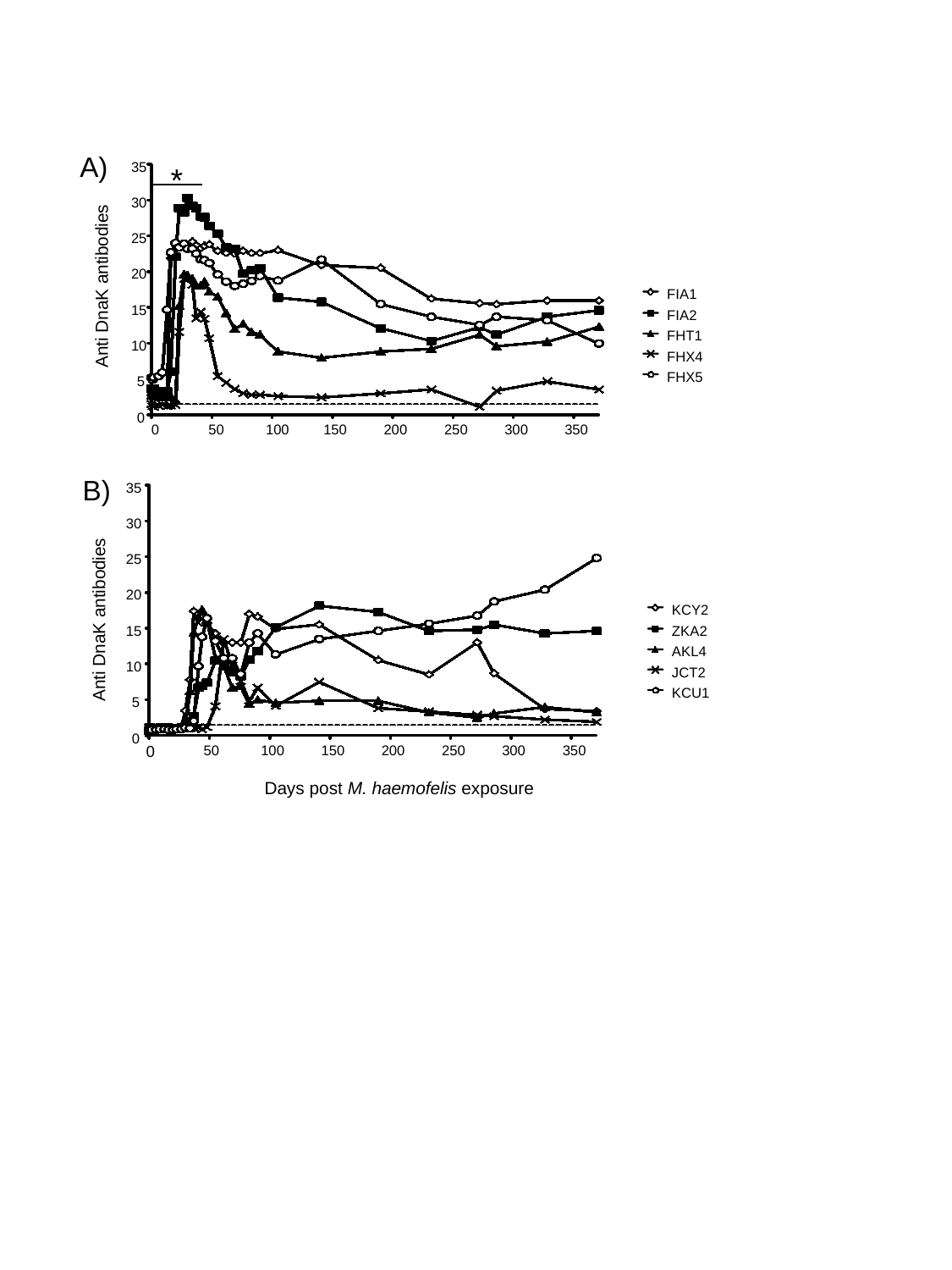

A)
35
30
25
20
15
10
5
0
0
50
100
150
200
250
300
350
Anti DnaK antibodies
FIA1
FIA2
FHT1
FHX4
FHX5
*
B)
35
30
25
20
Anti DnaK antibodies
KCY2
ZKA2
AKL4
JCT2
KCU1
15
10
5
0
0
50
100
150
200
250
300
350
Days post M. haemofelis exposure
